# Bioinformatics analysis and experimental validation of *C6orf120* as a potential prognostic marker and therapeutic target for liver hepatocellular carcinoma

**DOI:** 10.17305/bb.2024.11246

**Published:** 2024-10-10

**Authors:** Yingying Lin, Xin Wang, Yanyan Li, Xinyu Cui, Na Zhu, Xin Li

**Affiliations:** 1 Center of Integrative Medicine, Peking University Ditan Teaching Hospital, Beijing, China; 2 Center of Integrative Medicine, Beijing Ditan Hospital, Capital Medical University, Beijing, China

**Keywords:** *C6orf120*, liver hepatocellular carcinoma, prognosis, immune, angiogenesis, tumor microenvironment

## Abstract

The *C6orf120* gene is a novel gene whose function has not been fully defined. Previous studies have associated it with various liver pathologies, but its specific role in liver hepatocellular carcinoma (LIHC) remains unclear. This study aimed to investigate the diagnostic and prognostic value of *C6orf120* in LIHC, as well as its potential biological functions. In this preliminary research, we utilized data from various databases and bioinformatics tools, including TCGA, GEO, TIMER2, HPA, GEPIA, Linkeomics, Metascape, CIBERSORT, TargetScan, DIANA-microT, RNAinter, and ENCORI, to analyze the expression patterns and mechanisms of *C6orf120* in LIHC. Our bioinformatics analysis revealed that *C6orf120* is upregulated in LIHC and may serve as a diagnostic and prognostic biomarker. The aberrant expression of *C6orf120* in LIHC was further supported by clinical samples and cell lines. *In vitro* experiments demonstrated that the knockdown of *C6orf120* in HepG2 cells significantly reduced migration capacity without affecting proliferation. Additionally, the downregulation of *C6orf120* in LIHC cells appeared to inhibit endothelial cell migration and angiogenesis, which are critical in tumorigenesis and development. In conclusion, our findings suggest that *C6orf120* could serve as a novel diagnostic and prognostic biomarker for LIHC and is expected to be a prognostic marker and a potential therapeutic target in the clinical management of LIHC.

## Introduction

Liver cancer is one of the most aggressive malignancies in the digestive system [1], with liver hepatocellular carcinoma (LIHC) being its most common histological type [2]. Unfortunately, the prognosis of LIHC is generally poor, with a high mortality rate that nearly matches its incidence [2, 3]. LIHC progresses rapidly and is often asymptomatic in its early stages, resulting in late diagnoses and difficulties in applying conventional treatments, such as surgical removal and localized intervention [4]. Early detection is crucial for improving survival rates and the effectiveness of treatments. Despite significant advancements in LIHC diagnosis and treatment over recent decades, reliable diagnostic markers for early detection remain lacking. As a result, there is an urgent need to identify new biomarkers that can facilitate early diagnosis and enhance therapeutic outcomes for LIHC.

C6ORF120 is a novel secreted glycoprotein that was first characterized by our research group [5]. It has been implicated in the pathophysiology of several liver conditions, including autoimmune hepatitis [6], acute liver injury [7], and liver fibrosis [8]. Additionally, C6orf120 is involved in regulating immune cell functions, such as CD4 T-lymphocyte apoptosis [9], NKT cell activation [10], and macrophage polarization [11]. Given the intricate relationship between LIHC progression, underlying liver pathologies, and immune responses, exploring the potential association between LIHC and *C6orf120* is of significant interest. Our research group aims to bridge this knowledge gap.

In this study, we examined the expression patterns and possible mechanistic roles of *C6orf120* in LIHC using multiple databases. We also validated these findings through clinical sample analysis and *in vitro* experiments to further elucidate *C6orf120*’s role in LIHC. Our results suggest that *C6orf120* could serve as a promising biomarker for diagnosis and a potential therapeutic target for LIHC.

## Materials and methods

### Gene expression analysis

We used the Tumor Immune Estimation Resource 2.0 (TIMER2) database (http://timer.cistrome.org/) [12] to investigate the expression profiles of *C6orf120* across different cancer types. The tissue-specific distribution of *C6orf120* was analyzed using the Human Protein Atlas (HPA) database (http://www.example.com) [13], which also provided immunohistochemical staining data for *C6orf120* in LIHC tissues. We retrieved gene expression arrays and corresponding clinicopathological information from The Cancer Genome Atlas (TCGA) database (374 cancerous tissues and 50 normal liver tissues) [14]. The RNAseq data were converted to transcripts per million (TPM), and log2 (TPM + 1) was used in subsequent analyses. Additionally, we used two independent LIHC datasets for validation: GSE14520 (225 cancerous tissues and 220 normal liver tissues) and GSE76427 (52 cancerous tissues and 115 normal liver tissues).

### Clinical samples acquisition

LIHC tissues and adjacent normal tissue samples were collected from Beijing Ditan Hospital. The diagnostic criteria for LIHC were strictly followed according to the “Guidelines for the Diagnosis and Treatment of Primary Liver Cancer (2022 Edition)” [15]. The exclusion criteria were as follows: (1) patients under 18 years of age; (2) patients with a history of active or suspected malignant tumors in other organ systems within the past five years; (3) patients with secondary hepatocellular carcinoma or those who had previously received treatment for the condition; and (4) patients with prior diagnoses of autoimmune liver disease, drug-related liver disease, alcoholic hepatitis, cirrhosis, viral hepatitis, or HIV infection.

The tissue samples were processed and stained using standardized immunohistochemistry (IHC) protocols to compare C6ORF120 protein expression levels. Rabbit polyclonal C6orf120 antibody (Invitrogen, PA5-58864) was used at a 1:200 dilution. To assess systemic C6orf120 expression, we enrolled 40 LIHC patients and a control group of 30 healthy volunteers. Serum C6orf120 concentrations were measured using a commercially available ELISA kit (mlbio, China, YJ290341).

This study was reviewed and approved by the Ethics Committee of Beijing Ditan Hospital (No. DTEC-KT2024-002-01). The use of human tissues in this study complied with the ethical standards outlined in the Declaration of Helsinki. All patient data were anonymized to ensure confidentiality.

### Survival analysis

The predictive capability of *C6orf120* for overall survival (OS) across various cancers was assessed using the Gene Expression Profiling Interactive Analysis 2 (GEPIA2.0) database (http://gepia.cancer-pku.cn/) [16]. To evaluate the prognostic significance of *C6orf120* expression in LIHC, we analyzed data from both the TCGA and GSE76427 databases. LIHC samples were divided into high and low *C6orf120* expression groups based on the median expression level of *C6orf120* in each respective database. Kaplan–Meier survival analyses were performed using the “survival” and “jskm” packages in R software. Cox proportional hazard regression models were employed to compute hazard ratios (HRs). Additionally, using the “survival” package in R, nomograms incorporating significant clinical parameters were developed, and calibration plots were created for validation. The predictive accuracy of the nomogram was measured by the concordance index (C-index).

### Genetic alteration analyses

The cBioPortal database (https://www.cbioportal.org/) [17] was used to explore genetic alterations of *C6orf120* across different cancers. The “Mutations” module examined genomic alterations in the *C6orf120* gene within two LIHC datasets: AMC, Hepatology 2014 (*n* ═ 231) [18] and TCGA, Firehose Legacy (*n* ═ 379). Using GEPIA 2.0, we identified genes with high co-expression with *C6orf120* based on their Pearson correlation coefficient (PCC). Genes with higher PCC values were considered to have greater similarity to *C6orf120*. We selected the top nine most co-expressed genes along with *C6orf120* for genomic mutation analysis. Additionally, the GSCALite tool (https://www.editorialmanager.com/jtrm/default1.aspx) [19] was used to compare genomic mutation differences between *C6orf120* and its co-expressed genes, as identified by the GEPIA2.0 database.

### Functional enrichment analysis

The GeneMANIA tool (http://www.genemania.org/) [20] was employed to construct visual networks showing protein–protein interactions (PPIs) and to provide functional insights into these interactions. In this network map, each node represents a protein, with the diameter of the node reflecting the strength of its interactions. Different node colors indicate the biological functions of the associated genes. Using LinkedOmics (http://www.linkedomics.org/) [21], we applied the “LinkFinder” tool to identify genes co-expressed with *C6orf120*, visualizing the results as heatmaps. The “LinkInterpreter” module was then utilized for gene set enrichment analysis (GSEA) to explore related pathways. Analysis parameters were set with a false discovery rate (FDR) threshold of less than 0.05 and a simulation count of 1000. The top 500 genes showing the strongest positive correlation with *C6orf120* were selected based on their correlation coefficients. These genes were further analyzed for functional enrichment using gene ontology (GO) and the Kyoto Encyclopedia of Genes and Genomes (KEGG) via the Metascape tool (www.metascape.org) [22] and visualized using the “ggplot2” package in R.

### Immunocyte infiltration analysis

The CIBERSORT algorithm was used to estimate the abundance of 22 different immune cell types in patients, stratified by high and low *C6orf120* expression levels [23]. Gene expression data from TCGA were analyzed using the CIBERSORT online tool (http://cibersort.stanford.edu/) with default parameters. Additionally, we used the Tumor Immune System Interaction Database (TISIDB) (http://cis.hku.hk/TISIDB/) [23] to examine the relationship between *C6orf120* and the tumor immune microenvironment (TIME), including tumor-infiltrating lymphocytes, immunocyte co-stimulatory molecules, and co-inhibitory markers across multiple cancers. The results were visualized in a heatmap.

### Prediction of the competitive endogenous RNA (ceRNA) network

To identify miRNAs that may be part of ceRNA networks, we used TargetScan (http://www.targetscan.org) [24], DIANA-microT (http://diana.imis.athena-innovation.gr/DianaTools/index) [25], and RNAinter (http://www.rnainter.org) [26]. For predicting and analyzing the target long non-coding RNAs (lncRNAs) of the identified miRNAs, we employed the ENCORI database (https://starbase.sysu.edu.cn/) [27]. These regulatory networks were visualized using Cytoscape [28].

### Cell culture

Normal human liver cells (L02), LIHC cell lines (MHCC97H, Huh7, HepG2, and HCCLM3), and human umbilical vein endothelial cells (HUVECs) were provided by the Institute of Infectious Diseases, Beijing Ditan Hospital. All cells were cultured in Dulbecco’s Modified Eagle Medium (DMEM; Gibco, Carlsbad, CA, USA) supplemented with 10% fetal bovine serum (FBS; Gibco, Carlsbad, CA, USA) and 1% penicillin/streptomycin (P/S). Cells were incubated at 37 ^∘^C in a humidified atmosphere containing 5% CO_2_.

### *C6orf120* knockdown

To block *C6orf120* expression in HepG2 cells, we designed and synthesized two small interfering RNAs (si*C6orf120*-1 and si*C6orf120*-2) along with a scrambled negative control siRNA (siCtrl). These siRNAs, along with the control, were transfected into the cells using jetPRIME Transfection Reagent (Polyplus, France), following the manufacturer’s recommended protocol. The antisense sequences of the three siRNAs were as follows: si*C6orf120*-1: 5′-GCGAGUUCGAGAUGAAGGUTT-3′; si*C6orf120*-2: 5′-GCAUCGGCGUCUAUGGACATT-3′; siCtrl: 5′-UUCUCCGAACGUGUCACGUTT-3′.

### Western blotting

Cells were lysed using RIPA Lysis Buffer (Beyotime, Shanghai, China) supplemented with 1 mM phenylmethylsulfonyl fluoride (PMSF) and phosphatase inhibitors. After a 1-h incubation on ice, the supernatant containing proteins was collected by centrifugation at 4 ^∘^C at 12,000 rpm for 20 min. Protein concentrations were quantified using the BCA Protein Assay Kit (Beyotime) by measuring absorbance at 562 nm with a microplate reader. Proteins were resolved by 10% SDS-PAGE and transferred to a polyvinylidene fluoride (PVDF) membrane (Millipore, Darmstadt, Germany) via wet transfer. After blocking with 5% skim milk for 1 h, membranes were incubated with primary antibodies against Beta-Actin (Proteintech, 60008-1-IG) and C6orf120 (Invitrogen, PA5-58864) overnight at 4 ^∘^C. The membranes were washed three times with Tris-Buffered Saline and Tween (TBST) for 10 min each, followed by incubation with horseradish peroxidase-conjugated secondary antibodies for 1 h at room temperature. Target protein bands were visualized using an enhanced chemiluminescence (ECL) detection system and analyzed with ImageJ software (National Institutes of Health, USA).

### Cell Counting Kit-8 (CCK8) assay

Cells were seeded into 96-well plates at a density of 5 × 10^3^ cells per well. After 0, 24, 48, and 72 h of incubation, 10 µL of the CCK8 reagent (Beyotime) was added to each well. Following a 1-h incubation at 37 ^∘^C, absorbance was recorded at 450 nm using a microplate reader.

### Wound healing assay

HepG2 and HUVEC cells were inoculated into six-well plates and allowed to reach 95% confluence. A linear wound was created using a 10 µL pipette tip. After removing debris and dead cells with phosphate-buffered saline (PBS), the cells were cultured in serum-free medium with the respective treatments. Photographs were taken using a light microscope at 0, 24, and 48 h. Wound closure rates were quantified using ImageJ software.

### Transwell assay

Cell migration was assessed via a transwell assay. Cells were starved for 12 h, then harvested with trypsin, resuspended in FBS-free medium, and seeded into small chambers at a density of 1 × 10^5^ cells per well. After 24 h of incubation, cells on the upper surface of the membrane were removed with a cotton swab, and cells that had migrated or invaded through the membrane were fixed with 4% paraformaldehyde and stained with 0.1% crystal violet. The number of cells that traversed the membrane was quantified using an inverted microscope.

### Tube formation assay

HepG2 cells with *C6orf120* knockdown and corresponding control cells were cultured in 2 mL of FBS-free medium in six-well plates for 24 h. The conditioned medium was then collected and stored at 4 ^∘^C. Matrigel (BD Biosciences, Mississauga, Canada) was added uniformly to 24-well plates (200 µL/well) and incubated at 37 ^∘^C for 60 min to solidify. HUVECs were collected and resuspended in the HepG2-conditioned medium, adjusted to 4.5 × 10^5^ cells/mL, and 250 µL of this suspension was transferred to each well in 24-well plates. After an 8-h incubation, the formation of tubular structures was observed using an inverted light microscope. Tubule lengths and the number of junctions were analyzed using ImageJ software.

### Statistical analysis

Statistical analyses were conducted using SPSS version 26.0 (SPSS Inc., Chicago, IL, USA), GraphPad Prism version 9 (GraphPad Software, La Jolla, CA, USA), and R Studio (version 1.0.136). Unpaired *t*-tests or Mann–Whitney *U*-tests were used to compare two groups. For comparisons involving multiple variables, one-way ANOVA with the Kruskal–Wallis test was performed. Categorical variables were analyzed using chi-square tests, Fisher’s exact test, or continuity-corrected chi-square tests. A two-tailed *P* value < 0.05 was considered statistically significant.

**Figure 1.**
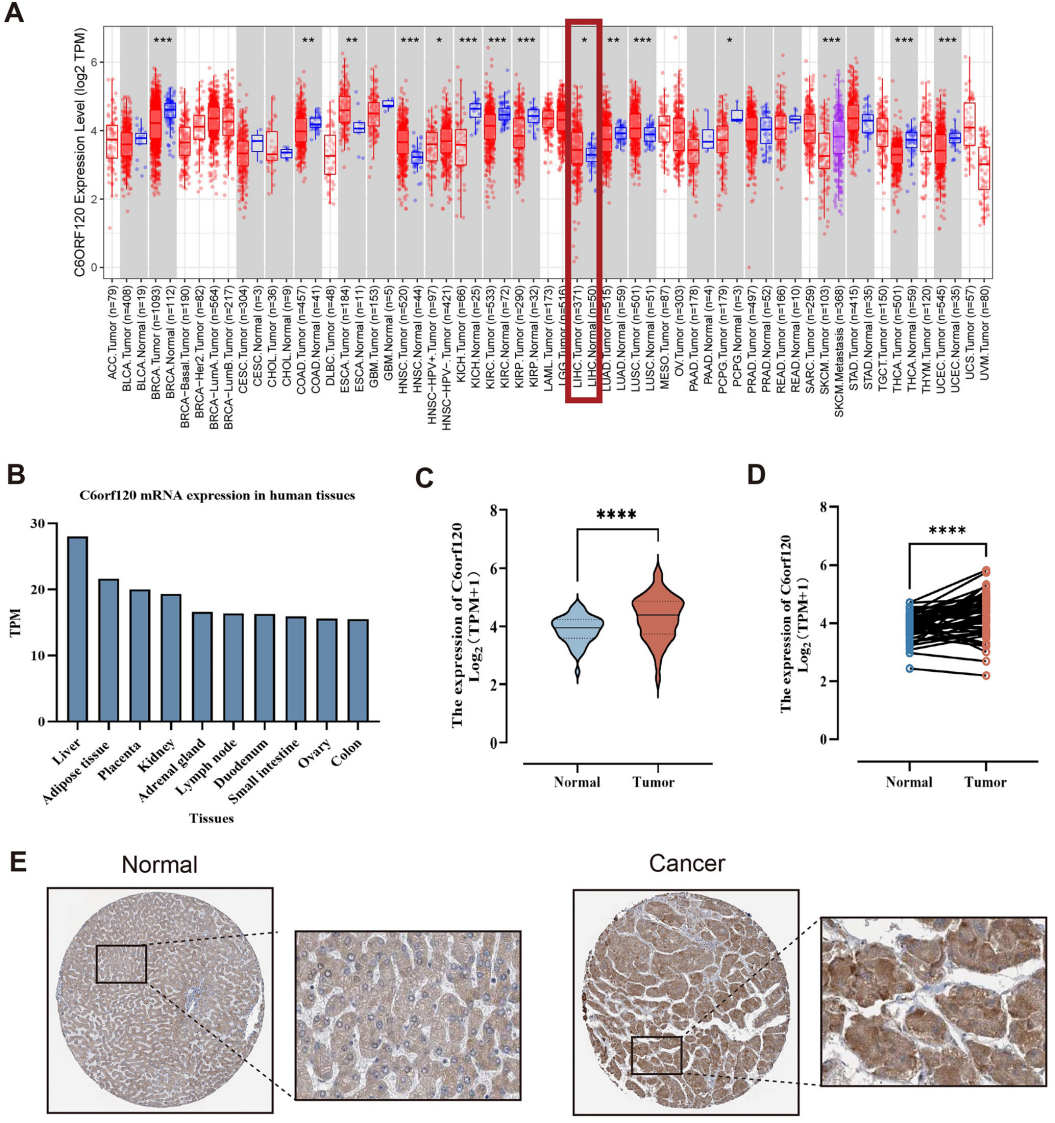
***C6orf120* expression landscape.** (A) *C6orf120* expression in different cancers from TIMER2.0. *P < 0.05; **P < 0.01; ***P < 0.001; (B) C6orf120 expression in different tissues from HPA; (C) Comparison of the expression of *C6orf120* in cancer and normal from the TCGA database; (D) Comparison of the expression of *C6orf120* in cancer and precancerous from the TCGA database; (E) The expression of C6ORF120 in LIHC from HPA. LIHC: Liver hepatocellular carcinoma; HPA: Human protein atlas; TCGA: The cancer genome atlas; TIMER2.0: Tumor Immune Estimation Resource 2.0.

## Results

### The abnormal expression of *C6orf120* in LIHC

The preliminary analysis of *C6orf120* gene expression across various human malignancies revealed a significant increase in *C6orf120* expression in multiple cancers, including esophageal carcinoma (ESCA), head and neck squamous cell carcinoma (HNSC), LIHC, and lung squamous cell carcinoma (LUSC) (*P* < 0.05) (Figure 1A). Tissue specificity analysis demonstrated that the human *C6orf120* gene is broadly expressed across 60 distinct tissues, with the liver exhibiting the highest expression levels (Figure 1B). Based on the TCGA database, *C6orf120* was overexpressed in LIHC samples compared to both normal liver tissues (Figure 1C) and paired adjacent non-tumoral liver tissues (Figure 1D). Similar findings were observed in the GSE14520 and GSE76427 databases (Figure S1).

**Figure 2.**
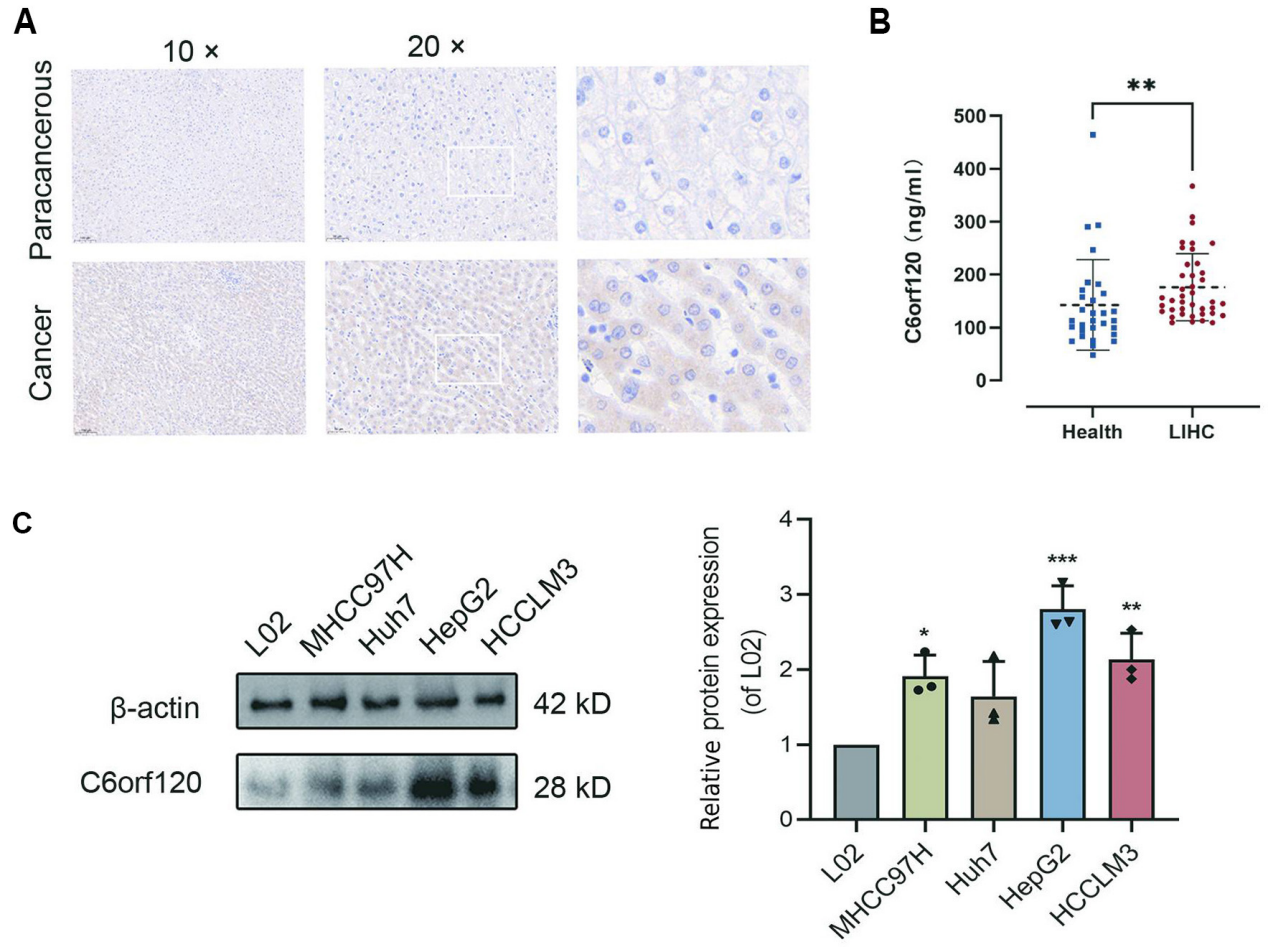
**Expression levels of the C6ORF120 in clinical specimens and cell lines.** (A) The expression of C6ORF120 in cancer tissue and paracancerous tissue of LIHC samples was detected by IHC; (B) Serum C6ORF120 levels in healthy controls and patients with LIHC; (C) C6ORF120 protein expression was detected in normal liver cells and LIHC cells. LIHC: Liver hepatocellular carcinoma; IHC: Immunohistochemistry.

At the protein level, data from the HPA database indicated markedly elevated C6ORF120 protein expression in cancer tissues (Figure 1E). Clinical tissue and blood samples validated this expression pattern. IHC revealed significantly higher C6ORF120 protein expression in tumor samples compared to adjacent normal tissues (Figure 2A and Figure S2). ELISA data showed elevated serum levels of C6ORF120 protein in LIHC patients compared to healthy controls (Figure 2B).

A Western blot assay confirmed the differential upregulation of C6orf120 in LIHC cell lines, including MHCC97H, HuH7, HepG2, and HCCLM3, relative to normal liver cells (L02). C6ORF120 expression in LIHC cells was 1.6–2.8 times higher than in L02 cells (Figure 2C). These results align with public database and clinical sample data, all of which indicate significantly elevated C6ORF120 expression in LIHC. Among LIHC cells, HepG2 exhibited the highest C6ORF120 expression, leading to its selection for subsequent knockdown experiments to investigate the function of *C6orf120*.

### High *C6orf120* expression was associated with clinicopathologic characteristics and poor prognosis in LIHC patients

The association between *C6orf120* expression and various clinical variables revealed asymmetric distributions of these parameters with increased *C6orf120* expression (Figure S3), suggesting its role in the pathogenesis of LIHC and underscoring its significance for further investigation.

**Figure 3.**
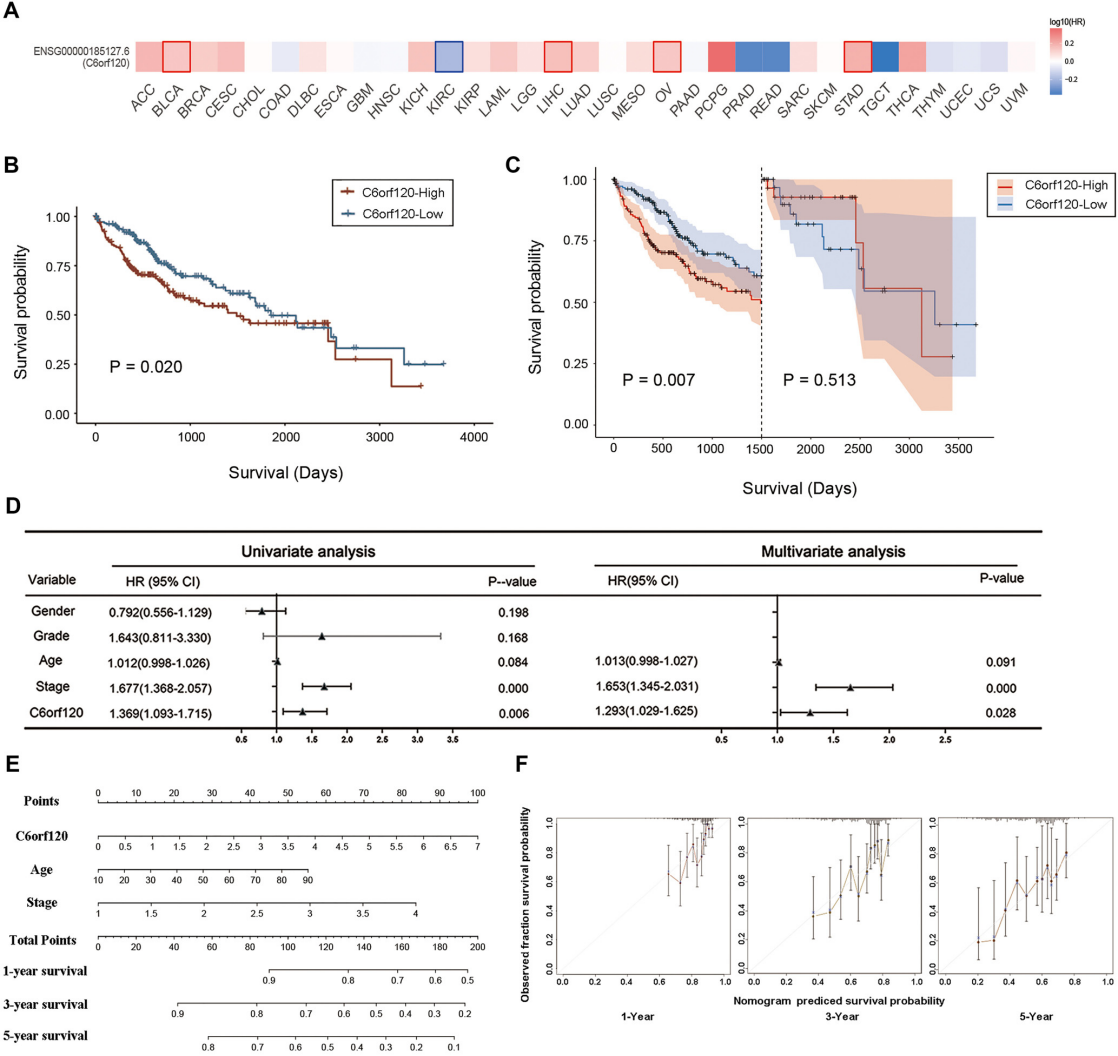
**Correlation between *C6orf120* expression and prognosis.** (A) GEPIA2.0 was used to analyze the effects of *C6orf120* gene expression on the patient’s prognosis in pan-cancer; (B) Kaplan–Meier survival curve showing a comparison of OS between patients with LIHC presenting high and low *C6orf120* expression in the TCGA cohort; (C) Landmark analyses (0–5 years) are shown; (D) Forest map showing univariate analysis and multivariate analysis about OS of LIHC patients in the TCGA database; (E) For patients with LIHC in the TCGA database, a nomogram based on grade, stage, and *C6orf120* was constructed to estimate the probability of 1-, 3-, and 5-year OS; (F) Nomogram calibration plots for determining the probability of OS at 1, 3, and 5 years. LIHC: Liver hepatocellular carcinoma; OS: Overall survival; TCGA: The cancer genome atlas.

*C6orf120*’s prognostic relevance was assessed across various cancer types, with notable findings in LIHC. In LIHC, an inverse relationship was observed between *C6orf120* expression and OS (Figure 3A). Pan-cancer analyses and Kaplan–Meier survival curves from the TCGA database identified *C6orf120* expression as a predictive factor for OS (Figure 3B) and five-year survival rates (Figure 3C). Cox regression analysis, incorporating variables with *P* values < 0.10 from univariate analysis (*C6orf120*, stage, and age), showed that *C6orf120* (HR ═ 1.293, 95% CI: 1.029–1.625, *P* ═ 0.028) was an independent prognostic factor in LIHC patients (Figure 3D). The C-index of the prognostic nomogram was calculated to be 0.655, indicating favorable discriminatory capability (Figure 3E). Calibration analysis further validated the nomogram’s accuracy in predicting survival probabilities (Figure 3F). The GSE76427 database also confirmed *C6orf120*’s prognostic significance (Figure S4).

### Analysis of genetic alterations in *C6orf120*

The genetic alteration landscape of *C6orf120* showed “deep deletion” as the predominant alteration type (Figure S5A). In LIHC, *C6orf120* had the highest frequency of “deep deletion” (Figure S5B). However, genetic alterations in *C6orf120* were identified in only 2.1% of the 610 LIHC patients analyzed (Figure S5C). Among nine analogous genes, *C6orf120* had the lowest mutation frequency, indicating its potential stability (Figure S5D).

### Function analysis of *C6orf120*-associated genes in LIHC

**Figure 4.**
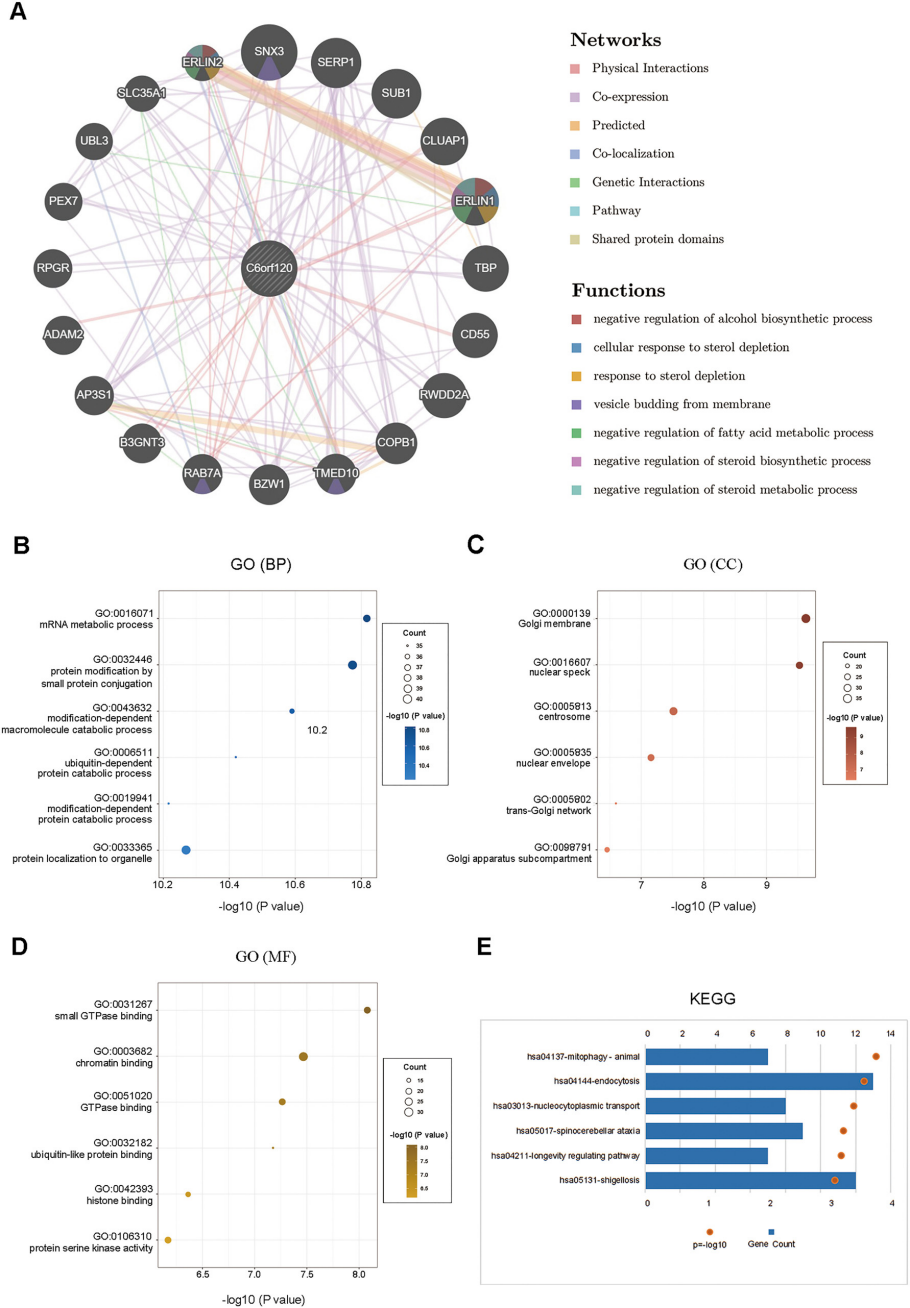
**Functional analysis of *C6orf120*.** (A) PPI network and functional analysis showing the gene set enriched in the target network of *C6orf120* (Gene-MANIA); (B–D) GO annotations *C6orf120* in LIHC include BPs, CCs, and MFs; (E) KEGG pathway analysis. PPI: Protein–protein interaction; MF: Molecular function; CC: Cellular component; BP: Biological process; GO: Gene ontology; KEGG: Kyoto encyclopedia of genes and genomes.

The PPI network, constructed using the GeneMANIA tool, revealed significant interactions between *C6orf120* and several key genes, including Snx3, Serp1, Sub1, Cluap1, and Erlin1. GSEA identified various biological functions for these interacting genes, such as the negative regulation of alcohol biosynthesis, cellular response to sterol depletion, vesicle budding from membranes, and negative regulation of fatty acid and steroid metabolic processes (Figure 4A).

**Figure 5.**
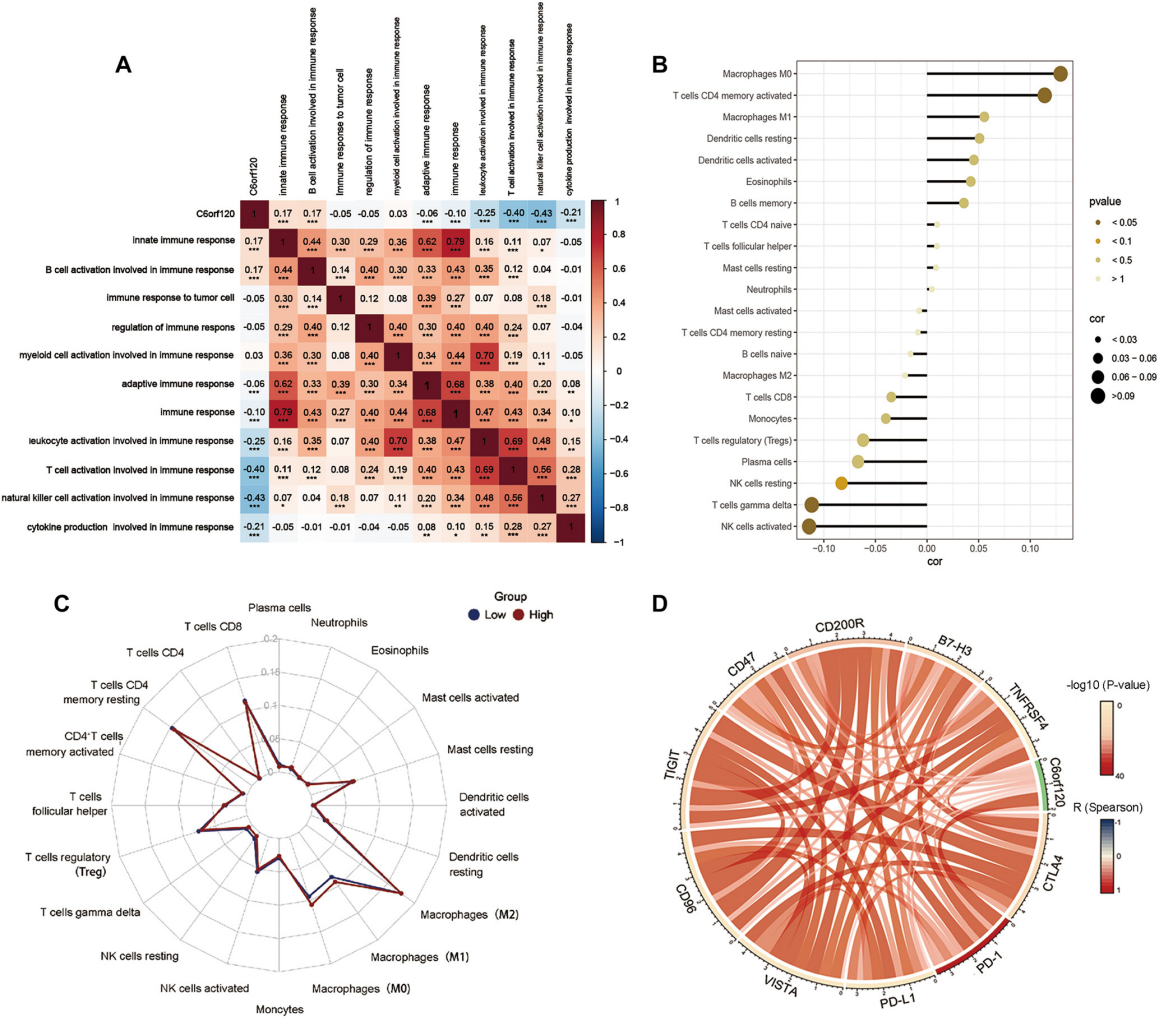
**Integrative analysis of *C6orf120* expression in the infiltrating immune microenvironment.** (A) Heatmap delineating the correlation of *C6orf120* and immune function enrichment scores. The red parts represented a positive correlation, while the blue parts represented a negative correlation (*P < 0.05, **P < 0.01, ***P < 0.001); (B) Correlation between expression of *C6orf120* and immune cell infiltration; (C) The radar plot displayed differential *C6orf120* expression on immune cell infiltration; (D) Correlation between *C6orf120* and inhibitory immune checkpoints. The width of the band represented the *R* value. The colors of the band represented the *P* value.

Using the Linkedomics tool, correlation analysis identified the top 50 genes most significantly correlated with *C6orf120* (both positive and negative correlations) (Figure S6A and S6B). GSEA analysis revealed that these co-expressed genes were involved in pathways, such as basal transcription factors, longevity regulation, and mRNA surveillance (Figure S6C).

The biological processes (BPs) most related to *C6orf120* included metabolic processes, protein modification by small molecule conjugation, and modification-dependent macromolecule catabolism (Figure 4B). Cellular components (CCs) associated with *C6orf120* included the Golgi membrane, nuclear speck, and centrosome (Figure 4C). Molecular functions (MFs) linked to *C6orf120* included small GTPase binding, chromatin binding, and GTPase binding (Figure 4D). KEGG pathway analysis showed that *C6orf120* was predominantly involved in mitophagy, endocytosis, and nucleocytoplasmic transport (Figure 4E).

Cluster analysis using Metascape indicated that genes associated with *C6orf120* were linked to mRNA metabolic processes and protein modification by small molecule conjugation (Figure S6D). GSVA analysis, conducted using the GSCA tool, showed a significant positive correlation between *C6orf120* and the androgen receptor (AR) and receptor tyrosine kinase (RTK) pathways, and an inverse correlation with the apoptosis and epithelial–mesenchymal transition (EMT) pathways (Figure S6E).

### The relationship between TIME and *C6orf120* expression in LIHC

Given the crucial role of immunity in tumor initiation and progression, evaluating the interaction between *C6orf120* and immune processes is essential. Our correlation analysis revealed a predominantly negative association between *C6orf120* expression and multiple immune functions, except for innate immune response and B cell activation (Figure 5A). To further clarify the role of *C6orf120* in tumor immunity, we analyzed its association with seven key immune cell types (B cells, CD8+ T cells, CD4+ T cells, M1 macrophages, M2 macrophages, neutrophils, and dendritic cells) in LIHC using the GEPIA database. The results, summarized in Table 1, showed a significant positive correlation of *C6orf120* with most of these biomarkers, except for HLA-DQB, which is associated with dendritic cells. These findings further underscore the role of *C6orf120* in the immune landscape of LIHC.

CIBERSORT analysis confirmed and quantified the associations between *C6orf120* expression and infiltration levels of 22 immune cell types. It revealed a positive correlation with M0 macrophages and activated memory CD4+ T cells and a negative correlation with activated NK cells and gamma delta T cells (Figure 5B). The radar plot comparison of immune cell infiltration between different *C6orf120* expression groups highlighted a significant upregulation in M0 and M1 macrophages in the high-expression group (Figure 5C). To explore the role of *C6orf120* in suppressing tumor immunity, we examined its association with inhibitory immune checkpoints (VISTA, CD96, TIGIT, CD47, CD200R, B7-H3, TNFRSF4, PD-1, PD-L1, and CTLA-4). *C6orf120* exhibited a positive correlation with these checkpoints (Figure 5D), particularly PD-1, suggesting its potential role in modulating immune responses in LIHC by suppressing immune activity. To provide a comprehensive view of *C6orf120* in the immune landscape of LIHC, we analyzed its correlation with tumor-infiltrating lymphocytes, co-stimulatory molecules, and co-inhibitory markers across various cancers using the TISIDB database. In each category, four primary outcomes were identified, with two showing positive correlations and two showing negative ones (Figure S7). Overall, *C6orf120* expression was negatively associated with most lymphocytes and immunomodulatory markers in LIHC.

**Table 1:** Correlation analysis between *C6orf120* and biomarkers of immune cells in LIHC

**Immune cell**	**Biomarker**	***R* value**	***P* value**
B cell	CD19	0.17	1.2 × 10^−3^ **
	CD79A	0.14	7.9 × 10^−3^ **
CD8+ T cell	CD8A	0.18	5.4 × 10^−4^ ***
	CD8B	0.11	2.7 × 10^−2^ *
CD4+ T cell	CD4	0.35	4.9 × 10^−12^ ***
M1 macrophage	NOS2	0.20	1.3 × 10^−4^ ***
	IRF5	0.25	1.4 × 10^−6^ ***
	PTGS2	0.29	2.4 × 10^−8^ ***
M2 macrophage	CD163	0.16	2.3 × 10^−3^ **
	VSIG4	0.25	1.2 × 10^−6^ ***
	MS4A4A	0.31	1.7 × 10^−9^ ***
Neutrophil	CEACAM8	0.11	3.0 × 10^−2^ *
	ITGAM	0.28	3.8 × 10^−8^ ***
	CCR7	0.18	3.9 × 10^−4^ ***
Dendritic cell	HLA-DPB1	0.20	8.5 × 10^−5^ ***
	HLA-DQB1	−0.01	9.2 × 10^−1^
	HLA-DRA	0.24	3.9 × 10^−6^ ***
	HLA-DPA1	0.27	8.0 × 10^−8^ ***
	CD1C	0.16	2.2 × 10^−3^ **
	NRP1	0.37	1.9 × 10^−13^ ***
	ITGAX	0.30	3.9 × 10^−9^ ***

*P value < 0.05; ** *P* value < 0.01; *** *P* value < 0.001.

### Construction of the *C6orf120*-associated ceRNA regulatory network

We initially predicted potential upstream miRNAs interacting with *C6orf120* using three bioinformatics tools: TargetScan, DIANA-microT, and RNAinter. This analysis identified 12 candidate miRNAs (Figure S8A). We then conducted expression correlation analyses using the Linkeomics platform. Based on the regulatory mechanisms of miRNAs, an inverse correlation was expected between *C6orf120* and its targeting miRNAs. As shown in Figure S8B, *C6orf120* was negatively correlated with hsa-miR-135b-5p, hsa-miR-1-3p, hsa-miR-27a-3p, and hsa-miR-20a-5p. However, only the correlation between hsa-miR-1-3p and *C6orf120* reached statistical significance, indicating that hsa-miR-1-3p could be a potential regulatory miRNA for *C6orf120* in LIHC (Table S1).

**Figure 6.**
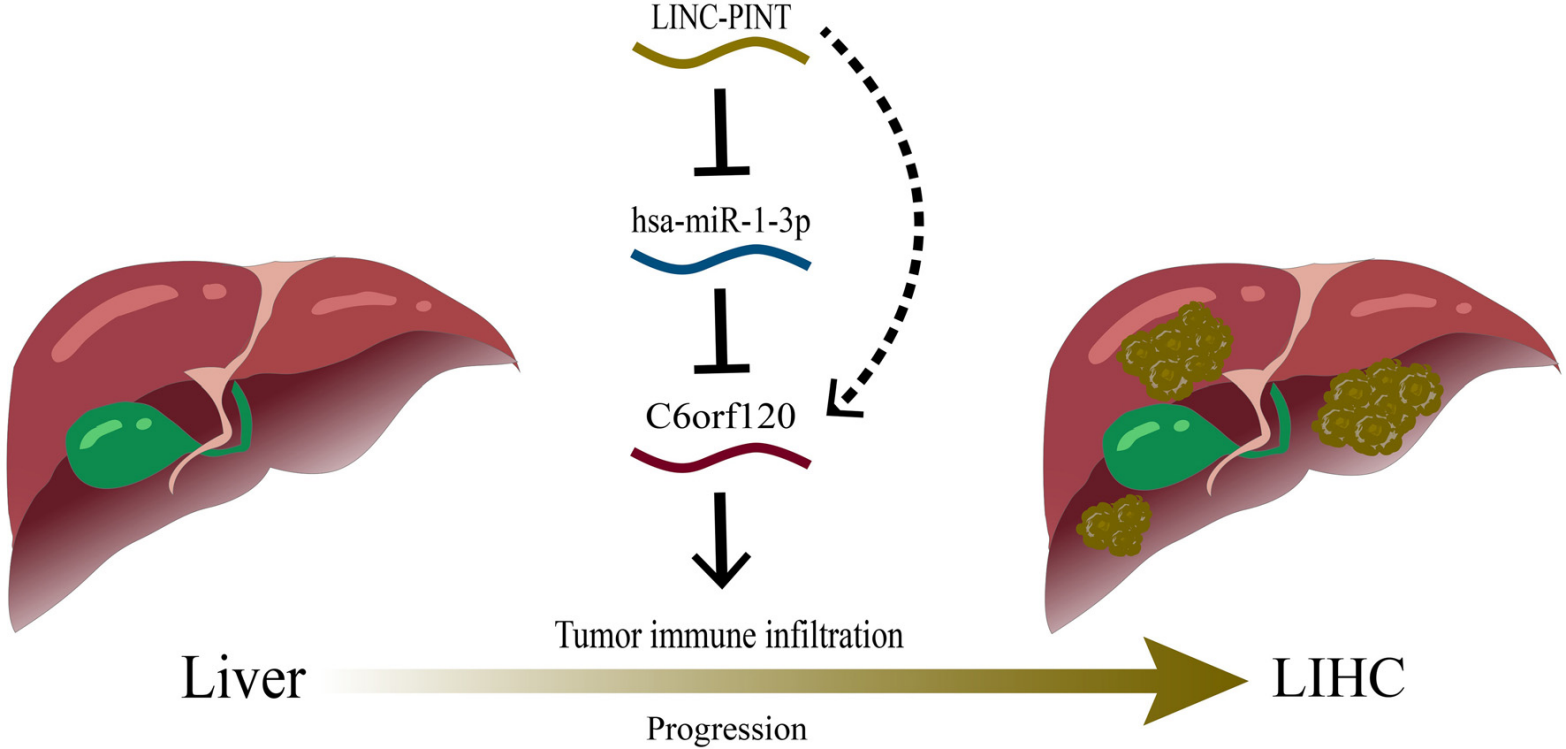
**The model depicting the LINC-PINT/hsa-miR-1-3p/*C6orf120* regulatory axis in LIHC tumorigenesis.** LIHC: Liver hepatocellular carcinoma.

Next, we identified 44 potential lncRNA candidates for hsa-miR-1-3p (Figure S8C). Among them, 21 lncRNAs were negatively associated with Hsa-miR-27a-3p, but only LINC-PINT showed statistical significance (Table S2). Using the ENCORI database, we found a positive correlation between LINC-PINT and *C6orf120* (Figure S8D), as well as a significant upregulation of LINC-PINT expression in LIHC tissues (Figure S8E). Taken together, the LINC-PINT/hsa-miR-1-3p/*C6orf120* axis may represent a potential regulatory pathway in the pathogenesis of LIHC (Figure 6).

### *In vitro* functional assay

**Figure 7.**
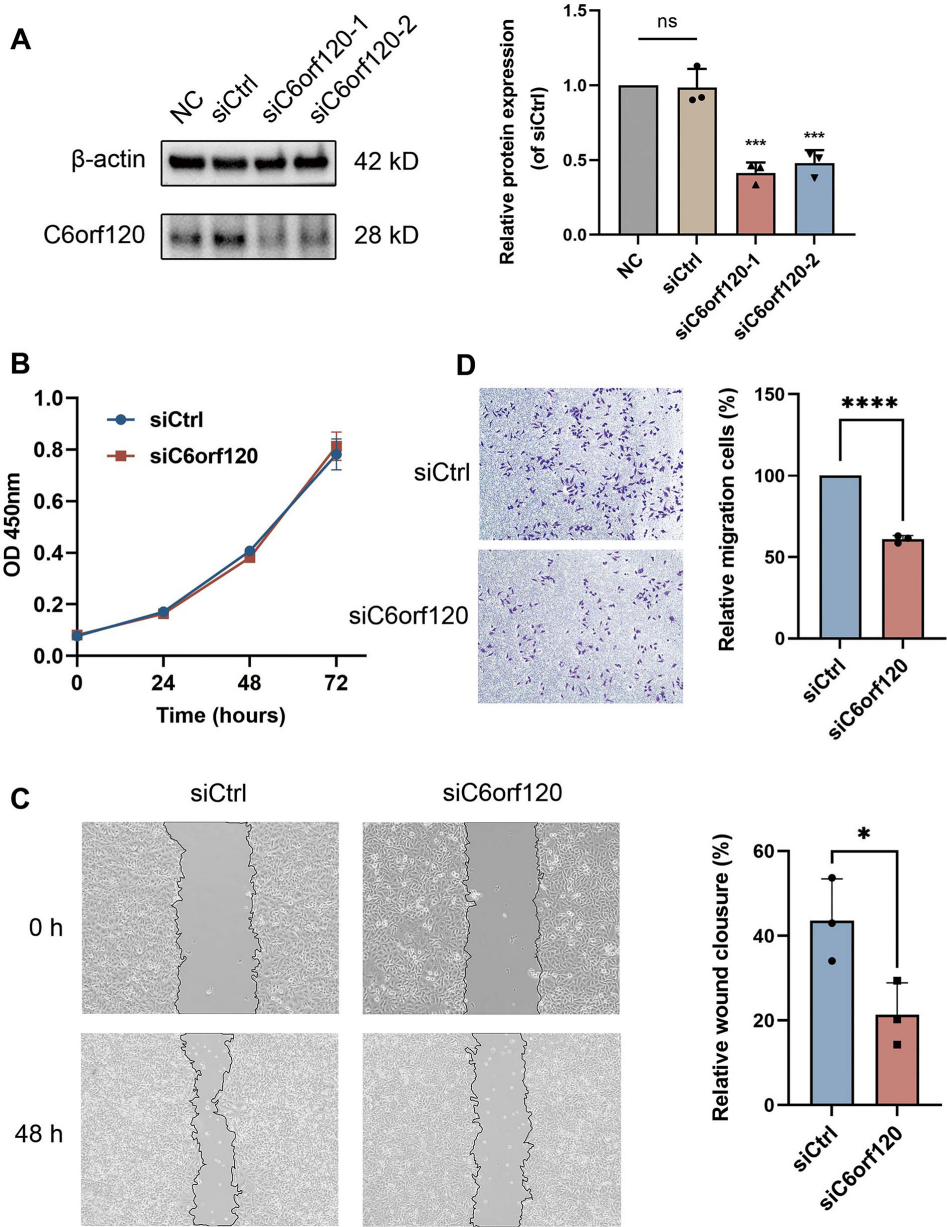
**Effect of *C6orf120* knockdown on LIHC proliferation and migration.** (A) The evaluation of transfection rate for silencing *C6orf120* by western blotting; (B) CCK8 assay was used to determine the viability of HepG2 cells at 24, 48, and 72 h after transfection with siCtrl or si*C6orf120*; (C) Comparison of the wound area in the *C6orf120* knockdown group and the control group after 48 h; (D) Comparison of the migrating cells siCtrl group between the si*C6orf120* group and the siCtrl group in the transwell migration assay. All experiments were performed three times and data are expressed as means ± standard. LIHC: Liver hepatocellular carcinoma; CCK8: Cell Counting Kit-8.

Western blotting was employed to evaluate C6ORF120 protein levels in HepG2 cells following siRNA transfection. The results showed that protein levels were significantly reduced in the si*C6orf120*-1 and si*C6orf120*-2 groups compared to the siCtrl and blank groups (*P* < 0.05) (Figure 7A). No significant difference was observed between the siCtrl and blank groups (*P* > 0.05), confirming the efficacy of our siRNA sequences. The si*C6orf120*-1 group exhibited the lowest C6ORF120 expression, indicating the highest knockdown efficiency. Therefore, si*C6orf120*-1 (si*C6orf120*) was selected for further experiments.

Based on these conclusions, we conducted additional analyses and *in vitro* experiments to explore the role of *C6orf120* in the biological characteristics of LIHC. The CCK8 assay was used to evaluate cell proliferation in all experimental groups. Our results showed no significant differences in absorbance values, which reflect cell proliferation, between the control and si*C6orf120* groups after 24, 48, and 72 h of culture (*P* > 0.05) (Figure 7B). These findings suggest that silencing *C6orf120* does not substantially impact the proliferative capacity of HepG2 cells under normal conditions.

Enhanced metastatic potential is a hallmark of tumor cells and is crucial in the progression and worsening of LIHC. To evaluate the role of *C6orf120* in HepG2 cell migration, we conducted wound healing and transwell assays. The wound healing assay revealed a significant reduction in migratory ability after *C6orf120* knockdown (Figure 7C). Consistently, the transwell assay showed a markedly decreased number of migrated cells in the si*C6orf120* group (Figure 7D). These findings suggest that *C6orf120* may modulate LIHC progression by influencing cell migration.

**Figure 8.**
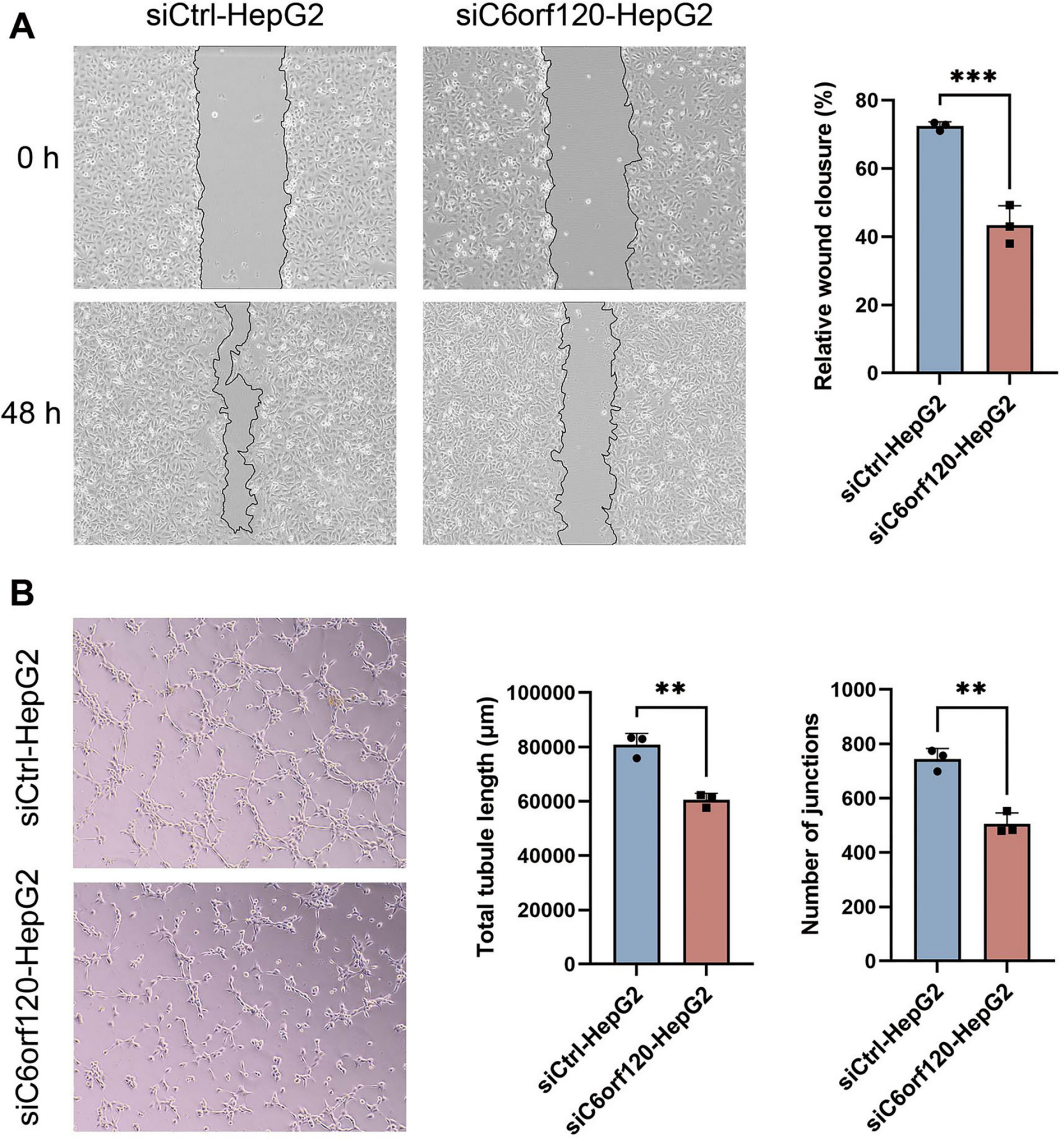
**Effects of *C6orf120*-knockdown HepG2 cells on the migration and angiogenic capacity of HUVEC.** (A) The impact of conditioned medium from si*C6orf120*-HepG2 on the migratory capacity of HUVEC; (B) The impact of conditioned medium from si*C6orf120*-HepG2 on the tube-forming capacity of HUVEC. All experiments were performed three times and data are expressed as means ± standard. HUVEC: Human umbilical vein endothelial cell.

Angiogenesis is a critical process linked to poor tumor prognosis and plays a significant role in LIHC metastasis [29]. To assess *C6orf120*’s impact on angiogenesis, we treated HUVECs with conditioned medium from HepG2 cells with and without *C6orf120* knockdown. We examined the effect of *C6orf120* knockdown on HepG2 cells’ ability to influence HUVEC migration and angiogenesis. The wound healing assay showed a significant reduction in HUVEC migratory capacity when treated with a conditioned medium from si*C6orf120*-HepG2 cells (Figure 8A), which is an important component of angiogenesis [30]. Similarly, the tube formation assay showed a substantial decrease in both the number of junctions and the length of tubules formed by HUVECs in the si*C6orf120*-HepG2 group compared to the control group (Figure 8B). These results suggest that *C6orf120* knockdown in HepG2 cells may attenuate HUVEC migration and angiogenesis via paracrine signaling mechanisms.

## Discussion

LIHC is acknowledged as a highly aggressive and lethal malignancy. Surgical and locoregional therapies often fail to satisfy the clinical needs of LIHC patients, and effective treatments remain to be developed [31]. Recent scientific projections reveal a rise in new diagnoses to approximately 1.4 million and deaths to 1.3 million by 2040 [32]. This anticipated increase highlights the urgent public health challenge of diagnosing and treating this disease. Elucidating the molecular mechanisms of LIHC carcinogenesis is crucial for identifying prognostic biomarkers and devising effective therapeutic strategies. The *C6orf120* gene exhibited significant expression in the liver and was correlated with an array of liver pathologies [6–10]. Given this association, we hypothesize that *C6orf120* may contribute to the pathogenesis of LIHC. This study explored the potential function of *C6orf120* in LIHC, with preliminary results indicating its potential as a promising biomarker and a novel target for therapy in LIHC patients.

Our preliminary data analysis indicated elevated *C6orf120* transcript and protein levels in LIHC tissues relative to normal controls. The high expression of C6ORF120 in the serum of LIHC patients highlighted its diagnostic potential as an easily accessible serum biomarker. Moreover, the significantly lower OS rate among LIHC patients with elevated *C6orf120* levels underscored its potential as a prognostic biomarker in LIHC. This observation prompted us to delve into the functional significance of *C6orf120* in LIHC.

The ceRNA regulatory network is crucial in cancer progression [33]. Hsa-miR-1-3p has been recognized for its tumor-suppressive properties, being downregulated in LIHC and capable of promoting apoptosis and suppressing cell proliferation when overexpressed in LIHC cells [34, 35]. Consistent with previous research, our investigation highlighted hsa-miR-1-3p as the most plausible candidate miRNA associated with *C6orf120*. Subsequently, we identified LINC-PINT as the most probable candidate lncRNA that could be upstream of the hsa-miR-1-3p/*C6orf120* axis. In line with the ceRNA hypothesis [36], LINC-PINT exhibited a positive correlation with *C6orf120* expression and a negative correlation with hsa-miR-1-3p. The regulatory axis of LINC-PINT-hsa-miR-1-3p-*C6orf120* may represent a newly identified pathway involved in the pathogenesis of LIHC. Additionally, the co-expression profile and regulatory factor network associated with *C6orf120* revealed robust interactions with several key proteins, including SNX3, SERP1, SUB1, CLUAP1, and ERLIN1. Several studies have supported the involvement of these molecules in regulating various tumor types [37–41]. Despite this, their correlation with LIHC remains unexplored. Therefore, investigating the interaction mechanisms between *C6orf120* and these molecules could yield valuable insights into the construction of a novel cancer regulatory network.

The immune system plays a crucial role in cancer etiology, progression, and therapeutic intervention [42]. In this study, we found a strong correlation between immune cell infiltration and *C6orf120* expression levels. As one of the most prevalent immune cells within the TIME, the polarization of macrophages into distinct functional phenotypes is a critical regulatory mechanism in tumor development [43]. M1 macrophages are classically characterized by enhancing T-cell immune responses [44], whereas M2 macrophages are known to secrete immunosuppressive factors that facilitate tumor cell proliferation [45]. However, the impact of macrophage polarization on tumorigenesis and progression varies across different studies. Our preliminary investigation suggests an association between *C6orf120* and macrophage polarization, favoring the M1 phenotype while suppressing the M2 phenotype. This paradoxical result may be explained by the complex role of macrophages, as macrophage polarization is not strictly binary and its effects are multifaceted, depending on the specific disease models and molecular interactions [46].

Immunotherapy has revolutionized cancer treatment, with approaches, such as checkpoint blockade, adoptive cell therapy, and cancer vaccines showing promising results across a spectrum of malignancies [47]. Our discovery that *C6orf120* can modulate the expression of immune checkpoint molecules implies that it could be a critical factor in developing novel immunotherapeutic strategies for LIHC.

The high mortality rate associated with LIHC is largely attributed to its metastatic potential, for which effective treatments remain elusive [48, 49]. Neovascularization within tumor tissues is a critical process that supplies nutrients essential for tumor metastasis [50]. Intense and rapid angiogenesis is a hallmark of malignant tumor progression and is associated with poor prognosis [51]. Clinically, inhibiting tumor angiogenesis within the tumor microenvironment (TME) is a well-established treatment strategy for LIHC [29]. Notably, our findings showed that the knockdown of *C6orf120* significantly impaired the migratory capacity of LIHC cells, and the conditioned medium from si-*C6orf120*-HepG2 cells potently inhibited angiogenesis. These results provide compelling evidence that *C6orf120* plays a key role in the regulation of angiogenesis in LIHC. By targeting *C6orf120*, it may be possible to inhibit the hematogenous metastasis of LIHC, offering a novel therapeutic strategy for managing the disease and improving patient outcomes.

Vascular endothelial growth factor A (VEGF-A) is a key driver of tumor angiogenesis [51]. Overexpression of VEGF-A not only fosters the phenotypic transformation of tumor endothelial cells but also leads to vascular immunosuppression, a significant barrier to the effectiveness of immunotherapeutic strategies [52]. Anti-VEGF therapies have been shown to enhance tumor immunity by reducing VEGF-mediated immunosuppression [53]. The combination of anti-angiogenic and immunotherapeutic approaches has yielded promising clinical outcomes. For example, the combination of bevacizumab and atezolizumab has emerged as a frontline therapy for LIHC, demonstrating superior efficacy compared to sorafenib [54, 55]. Our study suggests that *C6orf120* influences not only the immune landscape of LIHC but also the regulation of tumor angiogenesis, indicating that *C6orf120* could be a strategic target for an integrated approach to cancer therapy that encompasses both targeted therapy and immunotherapy. Furthermore, monitoring *C6orf120* levels may serve as an effective means of assessing treatment efficacy and predicting metastasis in LIHC. Therefore, it is crucial to investigate further the specific pathways and mechanisms through which *C6orf120* affects the TME.

However, our study encounters several limitations. Firstly, it relies on retrospective data obtained from public databases. To substantiate the clinical relevance of *C6orf120*, prospective clinical studies with the collection of additional clinical samples are required. Moreover, our current findings are based on *in vitro* cellular experiments, and further *in vivo* studies are essential to corroborate these results. Additionally, we have not yet identified and validated the specific downstream molecules of *C6orf120*. Future research will address these gaps to provide a more comprehensive understanding of the role and mechanisms of *C6orf120* in LIHC.

## Conclusion

In conclusion, our study preliminarily revealed the potential clinical value of *C6orf120* as a diagnostic and prognostic marker for LIHC patients. *C6orf120* may influence the immune response, promote LIHC metastasis, and modulate angiogenesis within the TME. These findings suggest that targeting *C6orf120* could open new avenues for therapeutic intervention in LIHC. Given its prognostic significance and potential as a therapeutic target, further research is warranted to explore the applicability of *C6orf120* in clinical practice.

## Supplemental data

Supplementary data are available at the following link: View of Bioinformatics analysis and experimental validation of *C6orf120* as a potential prognostic marker and therapeutic target for liver hepatocellular carcinoma (bjbms.org).

## Data Availability

The data underlying this article are available in the article and in its online supplementary material.
